# A continuity of midwifery care research mission, vision and agenda

**DOI:** 10.1186/s12913-026-14914-3

**Published:** 2026-06-09

**Authors:** Yvonne Kuipers, Alix Aitken-Arbuckle, Holly Jenkins, Kathryn Hardie, Amy Corrigan

**Affiliations:** 1 School of Health and Social Care, Edinburgh Napier University, Sighthill Court, Edinburgh, EH11 4BN Scotland, UK; 2 NHS Lothian, 51 Little France Crescent Old Dalkeith Road, Edinburgh, EH16 4SA Scotland, UK

**Keywords:** Continuity of midwifery care, mission, research strategy, research agenda, vision

## Abstract

**Background:**

Continuity of midwifery care is a priority within Scottish maternity services, yet limited evidence on key determinants and the absence of a shared research strategy constrain progress; therefore, this study aims to develop a systematic, evidence-based, and tailored process to inform a comprehensive Scottish continuity-of-midwifery-care research strategy, including its mission, vision, and aligned research agenda.

**Methods:**

Guided by implementation science, we engaged with 24 service providers, academics, managers, policymakers and service users’ advocates in Scotland. We collected data through an online poll, subgroup brainstorming sessions, plenary discussions, evaluations, video recordings, and research topic listings. Structural text reduction and a ranking and prioritisation process informed the synthesis and translation of the data into a research mission and vision. Interquartile ranking (Q_3_) was employed to select relevant and suitable guiding content elements for the mission and vision. The domains and determinants affecting the mission’s and visions’s functionality and capacity were framed using the Tailored Implementation for Chronic Diseases worksheets. Mean scores indicated their level of impact on the mission and vision. The ranked research topics were scored and prioritised based on their importance and relevance.

**Results:**

The research mission and vision were established. The capacity of change determinants, particularly the importance of necessary change and capable leadership, influence the capacity and operationalisation of the research mission and vision. Among the sixteen research topics, those focusing on economic evaluation, the organisation and management of continuity of care, and the future generation of continuity of midwifery care users and providers were ranked highest.

**Conclusions:**

We created a continuity of midwifery care research strategy and developed insights into embedding and applying this approach within the Scottish research context, guiding the operationalisation and further development of research on the continuity of midwifery care.

**Supplementary Information:**

The online version contains supplementary material available at 10.1186/s12913-026-14914-3.

## Background

Continuity of Midwifery Care (CMC) is a model of maternity care supported by global evidence demonstrating positive impacts on both the short- and long-term health and well-being of women and their families [1]. In CMC, the midwife acts as the lead professional in planning, organising, and delivering care to a woman from the first antenatal visit through to the postnatal period, within a multidisciplinary network of consultation and referral with other care providers [1, 2]. Since 2017, CMC has become a core element of Scotland’s maternity services policy. The Scottish Government’s five-year forward maternity and neonatal care plan, *The Best Start*, plays a key role in delivering CMC [3]. The report and its recommendations were developed through a research-to-practice synthesis and translation process, aiming to convert research knowledge into practical application [4–6]. To strengthen CMC’s evidence-based capacity, engagement from researchers, practitioners, and policymakers is essential to guide a research strategy and identify key areas most relevant to CMC research in Scotland [4–6]. To date, Scottish CMC research has primarily focused on initial implementation evaluation [7], but a socially responsible development and application of a research strategy or structured agenda within the Scottish CMC context is lacking. A research strategy could help shift from a reactive to a proactive research focus by identifying what research is needed, its anticipated form, and the questions that need answers. Furthermore, there is limited evidence regarding the moderating or mediating effects of specific contextual factors and determinants on the successful implementation of a CMC research strategy, which impedes the effective integration of CMC policy, practice and science [8, 9].

A mission and vision for a Scottish CMC research strategy encapsulate the core intentions, aspirations, values, beliefs, and sustainable practices of CMC research, while clarifying the future direction and valorisation of CMC research projects and studies. A clearly defined research mission and vision can communicate the direction and purpose of CMC research in Scotland, facilitating valorisation, identifying specific practices, stakeholders and establishing links between research and practice. When incorporating relevant and appropriate guiding content elements, a CMC research mission and vision can act as a cornerstone and unifying focal point, energising efforts in CMC research, providing structure and both short- and long-term planning, and fostering research collaboration [10, 11]. The mission and vision of CMC research establish a solid foundation for selecting research topics and developing a coherent research agenda. Well-crafted and thoughtfully constructed mission and vision statements can be powerful, often increasing the likelihood of successfully achieving the desired research strategy [10], ultimately leveraging knowledge to improve maternal and neonatal outcomes and experiences.

Our pre-implementation steps, which outline a systematic approach to developing a CMC research strategy, are described elsewhere [5]. In this paper, we focus on the subsequent phase of knowledge creation through synthesis and distillation, which involves retaining essential features or components, eliminating non-essential findings, and translating and refining this knowledge into a CMC research mission, vision, and priority topics for the research agenda. This process supports the development of a meaningful and stakeholder-informed CMC research strategy [6, 10]. Drawing on a comprehensive reservoir of insights from informants with context-specific CMC expertise, we aim to position the research strategy in alignment with Scottish needs to advance understanding of the CMC research context [12, 13]. Given the limited evidence on how key determinants influence the implementation of CMC research plans and the absence of a shared CMC research strategy, there is a need to align research needs and priorities with a structured agenda, including clear goal setting and iterative revision, to enhance the likelihood of successful operationalisation.

## Methods

### Aim

This study aims to develop a systematic, evidence-based, and tailored process to guide the creation of a comprehensive Scottish CMC research strategy, including its mission, vision, and aligned research agenda. Specifically, the objectives are to: (1) identify the key guiding content elements required to develop a CMC research mission and vision; (2) identify the dimensions and determinants shaping the functionality and capacity of the CMC research mission and vision; and (3) identify pertinent research topics.

### Design

Implementation science guided our work. We aimed to engage with real-world populations, reflecting actual conditions and the diverse interacting CMC contexts or settings in Scotland, which included sharing CMC interests, knowledge, and experiences [14, 15]. Implementation research involves informing and advising decision-making, promoting the identification, synthesis, and translation of stakeholder information, and influencing the direction and design of agendas and strategies [15–17].

### Informants

We aimed to represent stakeholders’ perspectives, such as practitioners, academics, policymakers, We considered managers, advocates for maternity service users, and (childbirth) educators as our group of informants. While no specific inclusion or exclusion criteria were set, we regarded informants as individuals with expertise, knowledge, and interest in Scottish CMC. We sought our informants in Scotland. To be eligible, informants had to self-identify as having an interest, knowledge, and/or expertise related to CMC in a broad sense (e.g., practice, management, academia, politics), but we did not specifically seek proponents or opponents of CMC. Informants were initially identified through the authors’ networks, which included practising midwives, lecturers, researchers, policymakers, and Health Board managers. The authors organised targeted events to facilitate multi-actor dialogues and sent invitational emails to inform potential informants about upcoming activities. The details of these activities are described elsewhere [5]. Recipients were asked to pass on the invitation (snowballing) and could email the first author to express their interest. If they wished, informants could also provide their email addresses for evaluation and validation related to mission and vision.

### Data collection

The data were collected in April 2024, with all authors involved in various activities and strategies to collect the data during a targeted CMC research event [5]. During the data collection sessions, we focused on the overarching questions: Why do we need CMC research? Why does CMC research need to exist? (mission). What do we want to achieve with CMC research? What purpose would CMC research serve? (vision). We used an online anonymous poll (Mentimeter©) where stakeholders could provide up to three answers to the question: What information on continuity of midwifery care is needed or missing? The responses were displayed as a word cloud during the event. During the events, stakeholders were invited to share and document their ideas, thoughts, and perceptions, and to write down actionable outcomes (brainstorming sheets) during interactive, semi-structured subgroup discussions (3 or 4 informants per subgroup). The brainstorming sheets were then shared and discussed during a plenary session. One researcher (HJ) took notes during the plenary session, focusing on verbalised sentiments about CMC. To develop a research agenda based on the stakeholders’ perceived importance, we asked them to discuss, list, and rank research topics, assigning first, second, third, and a wild card rank. The wild card was defined as a topic with qualities or characteristics that are indeterminate or unpredictable. This activity followed a procedure of brainstorming and discussion. The word cloud served as a means of inspiration and discussion, facilitating the formation of ideas. The stakeholders received a post-event evaluation form, which included an open-text box for adding suggestions or thoughts about CMC research.

### Synthesis and knowledge translation process

We focused on identifying, synthesising, distilling, and translating the data to inform our mission and vision. We merged the data from observation notes, brainstorming sheets, word clouds, transcripts, and evaluation forms into a single document. We removed identifiable information such as names and settings. We adapted the steps of the synthesis and translation process described by Thigpen et al. [6].

**Fig. 1:**
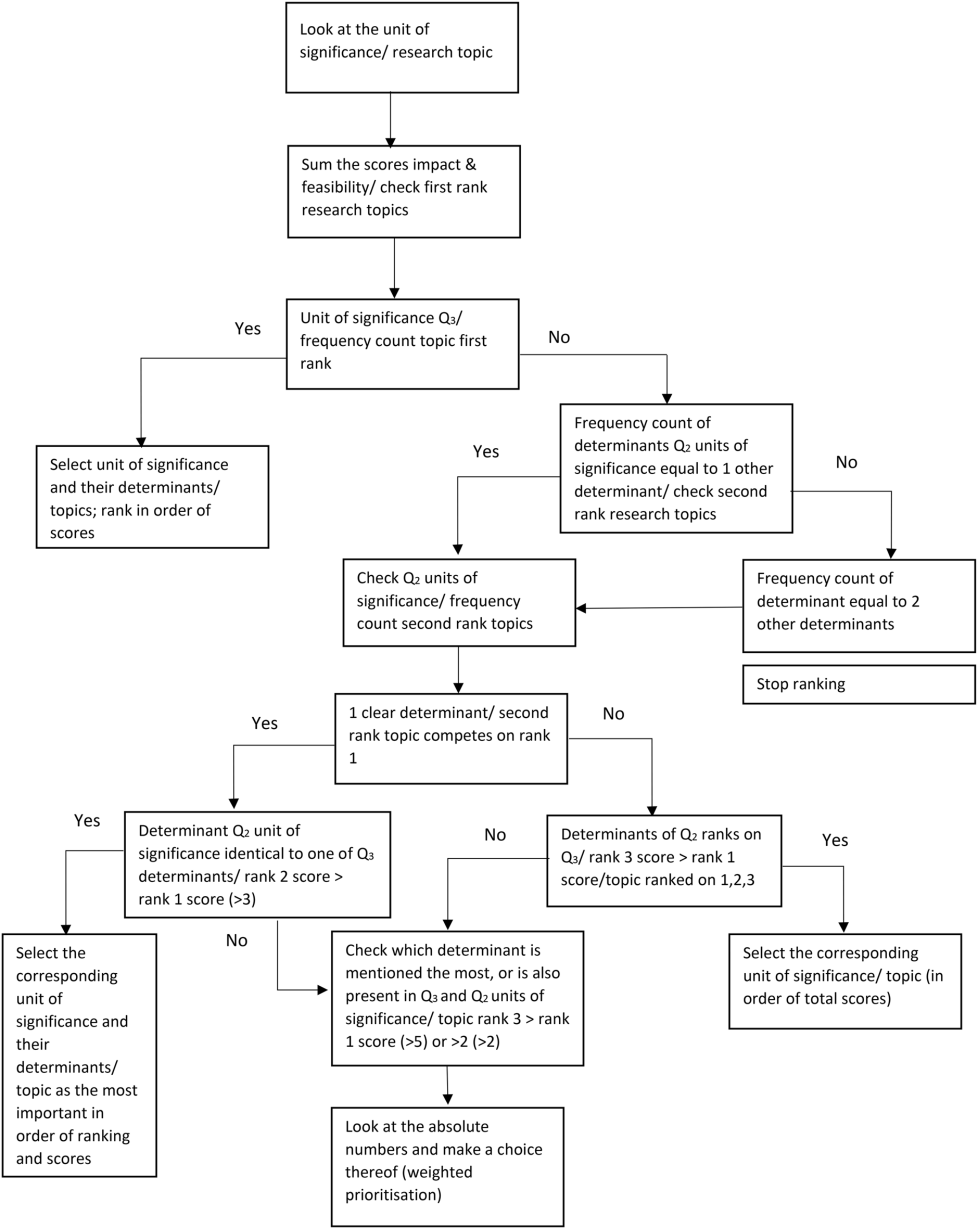
Flowchart data analysis prioritisation determinants mission, vision and research topics [5]

1. We reviewed the findings and categorised them based on relevance and appropriateness.
2. We applied structural text reduction: Line by line, three researchers (YK, AA-A, HJ) scrutinised the data for units of meaning (what is said in the original text fragments) and listed them.
3. The three researchers (YK, AA-A, HJ) converted the units of meaning into units of significance (what the text is talking about) by collaboratively rearranging, shortening, and restating the text, while maintaining the original meaning of the units of meaning [18]. We considered the units of significance as our key content elements for mission and vision [10]. We constructed an overview of the units of significance and added their barriers, facilitators and research recommendations when mentioned in the data (Additional Files 1 and 2).
4. Two researchers (YK, HJ) independently assessed and scored the impact, adherence, and feasibility of the units of significance [19] using the criteria and scores outlined in Box 1. The researchers based their scoring on their expertise and knowledge of CMC research, as well as midwifery experience, including caseload, community and hospital practice, and home birth [20]. Before scoring, YK and HJ discussed and acknowledged their potential biases to hold themselves and each other accountable during scoring and decision-making. The scores were compared, and the reasoning behind them was discussed, followed by a consensus-building discussion among all authors. We summed the scores (minimum-maximum score between three and 11) using the interquartile range (IQR) to select the most relevant and appropriate guiding content elements or units of significance that contribute to the mission and vision: lower (Q_1_), median (Q_2_), and upper (Q_3_) quartiles—Q_3_ representing the most relevant units of significance. A flowchart (Fig. 1) was used to guide the selection of key units of significance for formulating the mission and vision.
5. To identify and structure the context determinants that influence the functionality and capacity of the CMC research mission and vision [11], we employed the Tailored Implementation for Chronic Diseases (TICD) worksheets, a framework comprising 57 determinants grouped into seven domains: recommendations/factors of evidence, individual health professional factors, patient factors, professional interactions, incentives and resources, capacity for organisational change, and social, political, and legal factors [21]. YK and HJ re-examined the data [10, 11], and identified elements referring to TICD determinants, based on CMC evidence and their practice knowledge; a method recognised as a justified approach for TICD determinant selection [21]. Separate worksheets were used for the mission and vision elements.
6. To translate the findings for the end-user (i.e., everyone benefiting from CMC research), we employed an iterative process. We instructed an artificial intelligence chatbot (ChatGPT^©^) to assemble the units of significance into a mission and a vision statement. These statements were sent via Microsoft Forms to the informants, who were invited to comment on the function, focus, and form of the statements, as well as the quality and clarity of the narrative, specifically, the statements’ attainability, uniqueness, inspiration, and whether they depict a desirable future design for CMC research [10, 22].
7. We conducted a frequency analysis of the determinants explaining the mission and vision. We calculated the average of the determinants for each domain to determine their impact. We used the flowchart (Fig. 1) to identify the key determinants and domains that generate and maintain value for the CMC research mission and vision [10, 11].
8. We assigned scores to the ranked research topics: first rank = 3, second rank = 2, third rank = 1, and wild card = 2. Figure 1 helped list the research topics by importance.

**Box 1:** Criteria and scores

Impact: The likely impact of the unit of significance on the CMC research strategy (the impact if the unit of significance would be part of the mission and/or vision and thus research strategy)	
1 = No	
2 = Probably not	
3 = Uncertain	
4 = Probably	
5 = Yes	
Adherence: The likely impact of adherence to the unit of significance on the CMC research strategy (the level of seriousness of not including the unit of significance in the mission and/or vision/ non-adherence to the unit of significance)	
1 = minor impact on adherence	
2 = moderate impact on adherence	
3 = major impact on adherence	
Feasibility: The feasibility of the unit of significance in the targeted settings (how likely is research related to the unit of significance to contribute to real-world CMC)	
1 = low	
2 = moderate	
3 = high	

Priority setting was used to select the units of significance that are essential to the mission and vision, and to determine the order of the content in the strategy’s research agenda [13, 23]. Additional Files 1 and 2 show the domains, determinants, barriers, facilitators, and research recommendations of all units of significance. This paper reports the units of significance, their domains, and determinants.

## Results

### Informant group

A total of 24 informants participated, distributed across two CMC research events. The informants represented diverse regions of Scotland and included community-based, hospital-based, and homebirth midwives; NHS midwifery care managers such as lead midwives and directors of midwifery; policymakers from organisations including the Scottish Government, Royal College of Midwives, and Education Scotland; midwifery lecturers; early- and mid-career researchers; childbirth educators; and advocates for maternity service users. Some stakeholders held a single role, while others occupied multiple roles or positions. During the events, informants expressed sentiments about CMC, ranging from strong endorsement to apprehension and scepticism. Twenty-one informants provided email addresses to participate in the evaluation and validation of the mission and vision statements.

### Synthesis & translation CMC research mission and vision

#### CMC research mission

From 45 extracted units of meaning, we derived 23 units of significance (see Table 1), with a mean score of 9.08 (Standard Deviation ±1.47), interquartile range of Q_1_ = 8, Q_2_ = 9 and Q_3_ = ≥10. Eleven units of significance scored ≥10 (Q_3_) – representing the most relevant and appropriate contributions to the research mission. Two units of significance within the Q_2_ interquartile range shared the same determinant (priority of necessary change) as the ≥10 (Q_3_) units of significance. A total of 13 units of significance informed the CMC research mission. Twelve informants commented on the AI-drafted mission. After addressing comments regarding order, sentence spacing, and reduced wordiness, the CMC research mission was formulated as follows:

**Table 1:** Units of significance: research mission Continuity of Midwife Care (CMC)

Unit of Significance			Score
To contribute to philosophy of maternity care^*^			11
To amplify midwives’ voices^*^			11
To amplify women’s voices^*^			11
To ensure and sustain the CMC model^*^			11
To inspire transformational service change^*^			10
To treasure and add to the body of CMC knowledge^*^			10
To underscore the midwife’s essential role being with women^*^			10
To safeguard and champion the impact and value of CMC^*^			10
To nurture and support CMC midwifery and midwives^*^			10
To advocate for continued CMC successes^*^			10
To foster a dynamic CMC research culture^*^			10
To contribute to human reproductive rights^*^			9
To expand CMC awareness beyond midwifery profession^*^			9
To align with political/Governmental plans			9
To value midwifery and midwives in maternity services			9
To improve wellbeing for women and midwives			8
To take ownership of reproductive health			8
To have an impact on future generations of families and midwives			8
To safeguard the future of midwives and midwifery			8
To create long term impact on family health and wellbeing			8
To emphasise the impact of the midwife’s role in society			7
To improve the wellbeing of women, families and society			6
To act with integrity			6
Interquartile range: Q_1_ = 8, Q_2_ = 9 and Q_3_ = ≥10 ^*^Prioritised units of significance contributing to the CMC research mission according to flowchart			
**DOMAIN** ^**^	**Determinants (reported n)** ^**^	**Mean (SD ±)**	
Capacity for organisational change	Mandate, authority, accountability (6)/ Capable leadership (7)/ Relative strengths, supporters & opponents (3)/ Regulation, rules, policies (4)/ Priority of necessary change (8)/ Monitoring & feedback (2)	4.57 (± 2.43)	
Incentives & resources	Availability of necessary resources (3)/ Financial/ non-financial incentives/disincentives (3)/ Information system (5)/ Quality assurance & patient safety system (4)/ Continuing education system (4)/ Assistance for clinicians (1)	3.3 (±1.36)	
Individual health professional factors	Domain knowledge (5)/ Awareness & familiarity CMC recommendations (5)/ Knowledge own practice (5)/ Skills needed to adhere (3)/ Agreement with CMC evidence (3)/ Attitude towards CMC evidence (2)/ Expected outcome (3)/ Intention and motivation (4)/ Self-efficacy (3)/ Emotions (3)/ Nature behaviour (2)/ Capacity to plan change/ Self-monitoring or feedback (1)	3.15 (±1.28)	
Social, political & legal factors	Economic constraints on healthcare budget (3)/ Contracts (3)/ Legislation (2)/ Funder policies (2)/ Malpractice liability (3)/ Influential people (5)	3.0 (±1.09)	
Recommendations/factors of the CMC evidence	Strength CMC evidence (3)/ Quality CMC evidence (2)/ Clarity (2)/ Cultural appropriateness (2)/ Source CMC recommendation (2)/ Consistency with other CMC evidence (3)/ CMC recommendation feasibility (2)/ Effort (3)/ Trialability (2)/ Compatibility (2)/ Observability (1)	2.27 (±0.47)	
Professional interactions	Communication and influence (2)/ Team processes (2)	2 (±0)	
Patient factors	Patient needs (1)/ Patient beliefs & knowledge (2)/ Patient preferences (2)/ Patient motivation (2)/ Patient behaviour (1)	1.6 (±0.54)	

^**^Reported in relation to the 13 prioritised Units of Significance contributing to the CMC research mission

By fostering a dynamic research culture, we aspire to empower midwives to innovate, lead and champion the implementation of the Continuity of Midwifery Care (CMC) model and its lasting value.

We are committed to treasuring and expanding the body of CMC knowledge, broadening awareness of the CMC model beyond midwifery to influence maternity care systems.

We are dedicated to elevating the voices of midwives and women, while emphasising the core of the midwife’s role and philosophy of care: being with women. Through research, we aim to nurture and support CMC midwifery, safeguarding its lasting impact and advocating for its ongoing success.

Our mission is to drive transformative change in maternity care, ensuring the implementation and sustainability of the CMC model, promoting human reproductive rights, and advancing the philosophy of maternity care.

#### Dimensions and determinants CMC research mission

A frequency count identified 50 determinants associated with the 13 units of significance, influencing the functionality and capacity of the CMC research mission. Scores per determinant ranged from three to 11. The determinants were categorised into seven TICD domains: ‘Capacity for organisational change’ (7 determinants), ‘Incentives and resources’ (6 determinants), ‘Individual health professional factors’ (13 determinants), ‘Social, political and legal factors’ (6 determinants), ‘Recommendations/factors of the CMC evidence’ (11 determinants), ‘Professional interactions’ (2 determinants), and ‘Patient factors’ (5 determinants). ‘Priority of necessary change’, ‘capable leadership’, and ‘(organisational) mandate, authority and accountability’ had the greatest impact on the units of significance. These three determinants are part of the domain ‘Capacity for organisational change’ (Table 1).

#### CMC research vision

From 64 extracted units of meaning, we derived and scored 21 units of significance (see Table 2),

**Table 2:** Units of significance: research vision Continuity of Midwife Care (CMC)

Unit of significance			Score
Sustainable CMC as best practice and standard model of care^*^			11
Evidence model feasibility^*^			11
CMC motivation among midwives^*^			11
Identity statement CMC midwifery/ midwives^*^			11
Evaluation of the CMC model^*^			10
Effective leadership^*^			10
Research autonomy of midwives/ midwifery CMC domain^*^			10
Evidence model meaningfulness^*^			10
Positive (public) opinion CMC^*^			9
Open and positive CMC communication/ constructive dialogues^*^			9
Expand body of evidence^*^			9
Evidence model efficiency^*^			9
(Lifelong) CMC education^*^			9
Midwifery collegiality among CMC and non-CMC midwives			9
Renumeration midwives			9
Evidence long term outcomes maternal health			9
Evidence CMC appropriateness and effectiveness			8
Evidence model utility			8
Solidarity among midwives			8
Reflective practice			8
Paradigm transformation/ change			8
Interquartile range: Q_1_ = 8, Q_2_ = 9 and Q_3_ = ≥10 ^*^Prioritised units of significance contributing to the CMC research vision according to flowchart			
DOMAIN^**^	**DETERMINANTS (reported n)** ^**^	**Mean (SD ±)**	
Capacity for organisational change	Mandate, authority, accountability (5)/ Capable leadership (8)/ Relative strength of supporters and opponents (7)/ Regulations, rules, policies (3)/ Monitoring and feedback (7)/ Priority of necessary change (11)/ Assistance for organisational changes (2)	6.14 (± 3.1)	
Incentives resources	Availability necessary resources (3)/ Information system (7)/ Quality assurance and patient safety systems (3)/ Continuing education system (4)/ (Non)financial (dis)incentives (5)/ Assistance clinicians (2)	4 (±1.79)	
Individual health professional factors	Domain knowledge (6)/ Awareness & familiarity CMC recommendations (4)/ Knowledge own practice (4)/ Agreement CMC recommendation (2)/ Attitude towards CMC evidence/recommendation (3)/ Expected outcome (4)/ Intention & motivation (3)/ Self-efficacy (3)/ Nature of behaviour (2)	3.44 (±1.24)	
Recommendations/factors of the CMC evidence	Strength CMC evidence (7)/ Quality CMC evidence (4)/ Clarity (2)/ Cultural appropriateness (2)/ Source CMC recommendation (2)/ Consistency with other CMC evidence (2)/ CMC recommendation feasibility (1)/ Accessibility CMC (1)/ Effort (1)	2.44 (±1.94)	
Social, political & legal factors	Economic constraints healthcare budget (3)/ Contracts (1)/ Legislation (1)/ Influential people (4)/ Political stability (1)	2.0 (±1.41)	
Patient factors	Patient needs (3)/ Patient beliefs & knowledge (2)/ Patient preferences (2)/ Patient motivation (2)/ Patient behaviour (1)	2 (±0.7)	
Professional interactions	Communication and influence (2)/ Team processes (2)	2 (±0)	

^**^Reported in relation to the 13 prioritised Units of Significance contributing to the CMC research vision

showing a mean score of 9.33 (Standard Deviation ±1.03), interquartile range Q_1_ = 8.5, Q_2_ = 9 and Q_3_ = ≥10. Eight units of significance scored ≥10 (Q_3_) – indicating a relevant and appropriate contribution to the research vision. Five units of significance within the Q_2_ interquartile range shared the same determinant (priority of necessary change), which was identical to ≥10 (Q_3_) determinants of the units of significance. A total of 13 units of significance informed the CMC research vision. Twelve informants commented on the AI-drafted mission. After addressing the comments regarding order, length, wording and spelling, the CMC research mission was formulated as follows:

Our vision is a future where the Continuity of Midwifery Care (CMC) model is the gold standard in maternity care and is recognised as a beacon of best practice. This vision is driven by open and positive communication, fostering constructive dialogues that promote understanding, adoption, and ongoing improvement of the CMC model.

Our commitment is to expand the evidence base surrounding the CMC model, rigorously evaluating its efficiency, feasibility, and meaningfulness - empowering midwives to lead autonomously and strengthen their professional identity.

We aim to promote strong leadership and a positive perception of CMC among midwives, policymakers and the wider public - motivating (student)midwives and equipping them with the lifelong education they need to thrive in CMC.

#### Dimensions and determinants CMC research vision

A frequency count revealed 43 determinants alongside the 13 units of significance, shaping the functionality and capacity of the CMC vision, with scores ranging from 1 to 11 per determinant. Our research vision’s determinants were categorised into seven TICD domains: ‘Capacity for organisational change’ (7 determinants), followed by ‘Incentives and resources’ (6 determinants), ‘Individual health professional factors’ (9 determinants), ‘Recommendations/factors of the CMC evidence’ (9 determinants), ‘Social, political and legal factors’ (5 determinants), ‘Patient factors’ (5 determinants), and ‘Professional interactions’ (2 determinants). The four determinants most impactful on the units of significance were: ‘priority of necessary change’, ‘capable leadership’, ‘the relative strength of supporters and opponents’, and ‘monitoring and feedback’, all fitting the domain ‘capacity for organisational change’ and having the greatest influence. This was followed by ‘information system’, ‘strength of CMC evidence’, and ‘domain knowledge (health professional)’ (Table 2).

### Research topics

The participants proposed 16 distinct research topics and questions, receiving 99 votes. Each participant selected a first-choice topic, a second-choice topic, and a wildcard option, while 21 participants also indicated a third-choice topic. The ranking assigned by the participants yielded scores for 15 out of the 16 research topics, ranging from 0 to 41. Table 3 shows the order of importance and relevance of these topics, as determined by the participants. The highest-ranked topic, ‘Economic evaluation of CMC’, received a score of 41. Topics related to the organisation and management of CMC, the next generation of CMC users and providers, and women’s perceived CMC benefits and values scored between 12 and 22. Topics receiving a score of 10 or lower included, for example, care for specific groups of women, relational care, education, home birth, and providing care outside established guidelines.

**Table 3:** Ranked research topics

Research topics	Rank 1 score	Rank 2 score	Rank 3 score	Wild card score	Total score
Economic evaluation of CMC – cost benefit analysis which also includes wider societal benefits	27	4	0	10	41
Effective CMC team size achieving optimal outcomes for women and midwives – agree a benchmark	12	8	2	0	22
Understand new generation of women and midwives (‘TikTok’ generation) using and providing CMC and maternity services	0	0	0	20	20
What makes macro-level and mid-level managers amenable to change?	3	8	4	4	19
CMC midwives’ characteristics, attributes, strengths – who is the CMC midwife?	9	2	3	0	14
Essential components and assets for an effective CMC team	3	6	1	4	14
Benefits and values of CMC according to women	0	2	4	6	12
Clarification of the interrelated multidimensionality of relational care	0	8	2	0	10
Midwives’ experiences of providing outside of guidelines care within a relational continuity	6	0	0	2	8
The experiences of newly qualified midwives in providing CMC including home birth	3	2	1	2	8
Impact of education about midwifery values and (woman-centred) philosophy of care on CMC transition	6	0	0	0	6
Viability independent midwifery in Scotland/UK	3	2	1	0	6
Does team care offer the same level of relational continuity as caseload and how is this reflected in women’s satisfaction with care and pregnancy and birth outcomes?	0	2	1	0	3
The lived experiences of female refugees in Scotland and its implications for practice	0	2	1	0	3
Pregnant women with a BMI ≥30 kg/M^2^ receiving CMC	0	2	1	0	3
Time management CMC and professional boundaries	0	0	0	0	0
Total scores	72	48	21	48	189

Scoring: Rank 1 = 3 per vote; Rank 2 = 2 per vote; Rank 3 = 1 per vote; Wild card = 2 per vote

## Discussion

In this study, we used a systematic approach to align the expectations and objectives of the most critical CMC stakeholders. We outlined intentions and goals while envisioning achievements. We examined the necessary capacity, functionality, enablers, and challenges associated with operationalising the CMC research mission and vision. We developed an action plan for collecting and planning CMC research in Scotland. We crafted an inspiring and motivational CMC research mission and vision. The contextual determinants affecting the CMC research mission and vision reveal that the domain of ‘capacity for organisational change’ has the greatest influence on the functionality and capacity of our CMC strategy. Capacity for organisational change thus represents the needed and preferred direction that supports capacity building. It generates and sustains value in support of the CMC research mission and vision, and enhances the structure, transformation, and successful operationalisation of the CMC research strategy [10, 11, 24]. Several domain-specific determinants clarify the drivers of capacity for organisational change. These determinants shape the beliefs and attitudes of research stakeholders, including individual researchers, research groups, and funders, which are essential for changing and/or strengthening specific processes, research practices, performance, and structures [11, 24, 25]. The ‘priority of necessary change’ and ‘capable leadership’ were identified as central to the research mission and vision, emphasising their importance for CMC research intentions and intended achievements. Both factors highlight the need to elevate CMC on the agendas of political, healthcare research, and funding organisations, while emphasising leadership in conducting research and reorganising the structure of CMC research [13, 25]. Our research strategy provides a framework to achieve these aims. Determinants affecting the capacity for organisational change to maximise and realise the CMC mission and vision include the ‘relative strength of supporters and opponents,’ ‘monitoring and feedback,’ and ‘information system’—all of which can reduce social or cultural resistance to CMC research when these contextual determinants are strategically managed [26]. The domains and determinants exhibit a consistent relationship that guides the change methods of CMC research and how to adapt them to the Scottish context [27]. The prioritisation of research topics also aligns with the capacity for organisational change, particularly within the practice area, as the top-ranked topics focus on organising and managing CMC. The wildcard topic, ‘understand the new generation of women and midwives (‘TikTok’ generation) using and providing CMC and maternity services’, was ranked third, aligning with the need to anticipate how the uncertain future state of the CMC model may evolve for key stakeholders: childbearing women and midwives [28]. As a next step, in line with the domain of capacity for organisational change, the CMC’s mission, vision, and research agenda require widespread adoption. Funders must support the strategy by financing projects aligned with it, and researchers should select topics that reflect the CMC research strategy [29]. The economic evaluation of CMC received the highest ranking, potentially relating to the capacity for organisational change. Given the importance of women’s and midwives’ voices in the CMC research mission, we recommend identifying the quantitative and qualitative values, consequences, and effects relevant to women and midwives within the Scottish context [30]. Despite economic evaluation being ranked highest, the organisational, future-oriented, and perceived benefits and values of CMC concerning women’s topics may need to be investigated initially to identify Scottish CMC values, consequences, and effects, which will inform a locally tailored economic evaluation [31].

### Strengths and limitations

We employed a ranking and prioritisation process, involving managers and policymakers, to identify key units and research topics, enabling comparison between near-equivalent options and supporting practice-relevant priority setting [27]. While this approach may help bridge the research-practice translational gap and strengthen the relevance and uptake of the CMC research strategy [12, 13], it relies on judgment-based scoring, which introduces potential researcher bias. This risk is compounded by a possible pro-CMC orientation among self-identified expert participants, which may have shaped topic selection and interpretation. To mitigate bias, we included stakeholders with diverse professional backgrounds and perspectives, applied systematic frameworks for data structuring and synthesis, and ensured transparent, iterative deliberation processes [5]; however, residual bias cannot be excluded.

As no universally accepted method exists for mission- and vision-driven research prioritisation [32], our approach requires further validation and reliability testing (e.g., reproducibility of ranking across groups), although it contributes a novel step forward in this field [33]. The findings are context-specific to Scotland, which may limit transferability [27], though potentially generalisable elements reflect features of CMC internationally [34]. Additionally, this was a one-off priority setting, which may constrain long-term research responsiveness [13]; ongoing iteration, broader stakeholder inclusion, particularly childbearing women and student midwives and formal evaluation of strategy impact, functionality, and capacity are needed [5, 35]. Future work should assess how well the strategy is adopted by researchers, organisations, and funders, ensuring alignment with the evolving CMC landscape and long-term sustainability [11, 36].

## Conclusions

We designed and developed a comprehensive research mission, vision, and agenda for multi-actor CMC, synthesising and distilling its key features. Our study offers a strategic framework for CMC research, aiming to serve as a central reference point to catalyse efforts, structure short- and long-term planning, set direction and goals, and guide research as well as funding applications. Organisational change capacity, especially the ‘priority of necessary change’ and ‘capable leadership’, significantly influences the functionality and capacity of our CMC mission and vision. The sequence of the 16 research topics shapes the strategy’s research agenda, including economic evaluation, organisation and management of CMC, the future generation of CMC users and providers, and the perceived benefits and values of CMC - all recognised as urgent research priorities.

## Supplementary Information

Below is the link to the electronic supplementary material.

Supplementary Material 1: Additional File 1. Mission: Overview units of significance, TICD domains & determinants, impact & feasibility scores, barriers & facilitators, recommendations research strategies obtained from observation notes, flipcharts, word clouds, and evaluation forms.

Supplementary Material 2: Additional File 2. Vision: Overview units of significance, TICD domains & determinants, impact & feasibility scores, barriers & facilitators, recommendations research strategies obtained from observation notes, flipcharts, word clouds, evaluation forms.

Supplementary Material 3: Additional File 3. Standards for Reporting Qualitative Research (SRQR)

## Data Availability

The dataset supporting the conclusions of this article is included within the article (and its additional files).

## Abbreviations

CMC: Continuity of Midwifery Care
IQR: Interquartile range
SD: Standard Deviation
SRQR: Standards for Reporting Qualitative Research
TICD: Tailored Implementation for Chronic Diseases
