# Supplementary material for: A continuity of midwifery care research mission, vision and agenda
Source: BMC Health Serv Res. 2026 Jun 9;26:1097. doi: 10.1186/s12913-026-14914-3 (PMC13465298; doi:10.1186/s12913-026-14914-3)
Supplement: Supplementary file 1 [file 12913_2026_14914_MOESM1_ESM.pdf]

Additional file 1. Mission: Overview units of significance, TICD domains & determinants, impact & feasibility scores, barriers & facilitators, recommendations research strategies obtained from observation notes, flipcharts, word clouds, evaluation forms and transcripts research proposals explanations (adapted from Aakhus et al., 2014; Cane et al., 2012; Flottorp et al., 2013; Dunlop et al., 2023).

| **TICD domain** | **TICD determinant** | **Unit of significance to which the determinant applies** | **Likely impact of the determinant on CMC research**[1] | **Likely impact on adhering to the unit of significance**[2] | **Feasibility unit of significance**[3] | **Barriers and facilitators** | **Recommendations research strategies (e.g. specific clinicians, practices, populations, researchers, methodologies, funds)** |
|---|---|---|---|---|---|---|---|
| Capacity for organisational change | Authority, capable leadership, priority necessary change | **To inspire transformational service change*** | 5 | 3 | 2 | *Facilitators* Motivation, willingness, commitment from all stakeholders (government, NHS management, midwives) | |
| Social, political and legal factors | Economic constraints on healthcare budget, contracts, legislation, influential people | | | | | | |
| Factors from the CMC evidence | Strength CMC evidence | **To improve wellbeing for women and midwives** | 4 | 2 | 2 | *Facilitators* Prioritising women and midwives in healthcare | Public engagement |
| Individual health professional | Awareness and familiarity CMC recommendations, self-monitoring or feedback | | | | | | |
| Incentives and resources | Information system, quality assurance and patient safety system, continuing education system | | | | | | |
| Capacity for organisational change | Mandate, authority, accountability, capable leadership, priority of necessary change | | | | | | |
| Individual health professional factors | Attitude towards CMC evidence/recommendation, intention and motivation | **To take ownership of reproductive health** | 3 | 3 | 2 | *Barriers* Patriarchy and capitalism society | Public engagement. Addressing lived CMC experiences of |

| | | | | | | | |
|---|---|---|---|---|---|---|---|
| Patient factors | Patient needs | | | | | *Facilitators* Feminism. Prioritising women and midwives in health care. Needs assessment reproductive healthcare. Positive experiences CMC. Being up to date with political agenda | women and midwives. Challenge status quo maternity services in discussions (papers) |
| Incentives and resources | Availability necessary resources, (non)financial (dis)incentives, information system, continuing education system | | | | | | |
| Capacity for organisational change | Mandate, authority, accountability, capable leadership, relative strength supporters and opponents, regulation, rules, policies, priority of necessary change | | | | | | |
| Factors from the CMC evidence & | Effort (recommended behaviour) | **To treasure and add to the body of CMC knowledge*** | 4 | 3 | 3 | *Barriers* Lack of support of the CMC model, lack of commitment to CMC | |
| Social, political and legal factors | Economic constraints on the healthcare budget, funder policies | | | | | | |
| Factors from the CMC evidence | Consistency with other CMC evidence | **To contribute to Human rights/ reproductive rights*** | 4 | 3 | 2 | *Barriers* Reproductive rights not public health priority *Facilitators* CMC being a WHO recommendation. Prioritising women and midwives on the national healthcare agenda | Give women a voice/ actively involve women in set up/ development CMC research |
| Individual health professional factors | Attitude towards CMC evidence/ recommendation (cognition) | | | | | | |
| Incentives and resources | Quality assurance and patient safety system, continuing education system | | | | | | |
| Capacity for organisational change | Mandate, authority, accountability, capable leadership, regulation, rules, policies, priority of necessary change | | | | | | |
| Social, political and legal factors | Legislation, malpractice liability, influential people | | | | | | |
| Factors from the CMC evidence | Strength CMC evidence | **To have an impact on future** | 4 | 2 | 2 | *Facilitator* Sustainable goals adherence | |

| | | | | | | | |
|---|---|---|---|---|---|---|---|
| Individual health professional factors | Intention and motivation, self-efficacy, expected outcome (cognition) | **generations of families and midwives** | | | | | Making midwifery more visible - social media (campaign). Dissemination CMC research |
| Patient factors | Patient needs, patient beliefs and knowledge, patient preferences, patient motivation, patient behaviour | | | | | | |
| Capacity for organisational change | Priority of necessary change, monitoring and feedback | | | | | | |
| Individual health professional factors | Domain knowledge, awareness and familiarity with CMC recommendations, knowledge own practice, skills needed to adhere, intention and motivation, self-efficacy, expected outcome, nature of behaviour | **To contribute to philosophy of maternity care*** | 5 | 3 | 3 | *Barriers* Technology. Remuneration. Task-oriented care. Theory-practice gap. Prioritising the institution/ institutional philosophy. Patriarchal and technocratic ideologies in maternity services. Moving away from the true identity of midwifery/midwives. CMC is not standard of care/ practice *Facilitators* Shared CMC vision. General belief in CMC. Prioritising women and midwives in maternity services. Emphasize and acknowledge the | Place value on the woman's experience in CMC research. Epistemological approach to research. Study CMC ideations among (student) midwives. Position papers. Discussion papers. |

| | | | | | | | |
|---|---|---|---|---|---|---|---|
| | | | | | | specialised identity of midwives. Placing value on physiology. Value-based care. Woman-centred focus | |
| Individual health professional factors | Capacity to plan change (professional behaviour) | **To align with political/ Governmental plans** | 3 | 3 | 2 | *Barriers* Lack of shared CMC vision, lack of finance for CMC *Facilitators* Political agenda around CMC. Feminism. Discussing CMC with people in positions to affect change. Commitment to drive change from policy makers | Decisionmakers presenting their vision for maternity care. National surveys. |
| Incentives and resources | Availability necessary resources, information system, quality assurance and patient safety system | | | | | | |
| Capacity for organisational change | Capable leadership, relative strength supporters and opponents, priority of necessary change, monitoring and feedback | | | | | | |
| Social, political and social factors | Economic constraints healthcare budget, influential people | | | | | | |
| Individual health professional factors (cognition) | Agreement with CMC evidence, intention and motivation, emotions, nature of behaviour, capacity to plan change | **To amplify midwives' voices*** | 5 | 3 | 3 | *Barriers* Prioritisation of money, production, politics. Bullying, harassment around CMC in maternity services affecting midwives to freely voice supporting/ providing CMC *Facilitators* Feminism. Management respecting midwives | Asking midwives for their views and opinions in CoC research activities |
| Capacity for organisational change | Mandate, authority, accountability, capable leadership, priority of necessary change | | | | | | |
| Patient factors | Patient needs, patient beliefs and knowledge, patient preferences, | **To amplify women's voices*** | 5 | 3 | 3 | *Barriers* Silence/ stigma pregnancy, | Asking women for their views and opinions in CMC |

| | | | | | | | |
|---|---|---|---|---|---|---|---|
| | patient motivation, patient behaviour | | | | | childbirth, women's health *Facilitator* Feminism | research activities. Public engagement |
| Social, political and legal factors | Influential people | | | | | | |
| Factors from the CMC evidence | Strength CMC evidence | **To underscore the midwife's essential role being *with women**** | 5 | 2 | 3 | *Barriers* Midwifery in crisis. Influx in women wanting to birth outside guidelines and freebirth. CMC not standard model of care *Facilitator* Feminism | Position papers |
| Incentives and resources | Availability necessary resources, (non)financial (dis)incentives, information system, assistance for clinicians | | | | | | |
| Individual health professional factors | Agreement with CMC evidence, expected outcome, intention and motivation, self-efficacy (cognition) | **To value midwifery and midwives in maternity services** | 4 | 3 | 2 | *Barriers* Reduced to nurses working for obstetric team. Technology taking over. Payment. Reduced to a series of tasks. Shift patterns. Changing job roles. Midwives leaving *Facilitators* Management/ leaders having the wellbeing of midwives at heart. Wellbeing support mechanism in place. Mechanisms in place to support family life. Managers showing an interest in midwives | Study populations of student midwives, newly qualified midwives, midwives with caring responsibilities. |
| Availability resources | Availability necessary resources, (non)financial (dis)incentives, information system, assistance for clinicians | | | | | | |
| Individual health professional factors | Agreement with CMC recommendation, expected outcome, intention and | **To safeguard and champion the** | 4 | 3 | 3 | *Facilitators* Placing value on physiology. Reflexive practice | Capture impact of midwives in research. Capture the CMC |

| | | | | | | | |
|---|---|---|---|---|---|---|---|
| | motivation, self-efficacy (cognition) | **impact of CMC midwives*** | | | | | midwife's role and identity (social positioning, professional socialization). Capture woman-centredness (concept woman-centred care) |
| Capacity for organisational change | Mandate, authority, accountability, capable leadership, regulation, rules, policies, priority of necessary change, monitoring and feedback | | | | | | |
| Individual health professional factors | Domain knowledge, awareness and familiarity with CMC recommendations, knowledge own practice, expected outcome, emotions | **To nurture and support CMC midwifery and midwives** | 5 | 2 | 3 | *Barrier* Dissolving midwifery role. CMC not standard model of care *Facilitator* Working within professional boundaries/ true role | |
| Patient factors | patient beliefs and knowledge, patient preferences, patient motivation | | | | | | |
| Professional interactions | Communication and influence, team processes | | | | | | |
| Incentives and resources | Availability necessary resources, information system, quality assurance and patient safety system, continuing education system | | | | | | |
| Professional interactions | Communication and influence, team processes | **To expand CMC awareness beyond midwifery profession (management, policy, obstetrics)*** | 4 | 3 | 2 | *Facilitators* System wide support. Buy in from medical staff | |
| Incentives and resources | Availability necessary resources, information system | | | | | | |
| Capacity for organisational change | Priority of necessary change | | | | | | |
| Factors from the CMC evidence | Strength CMC evidence | **To create long term impact on family** | 4 | 2 | 2 | *Facilitators* Midwifery perceived as integral | Target specific (marginalized/ |

| | | | | | | | |
|---|---|---|---|---|---|---|---|
| Patient factors | Patient needs, patient beliefs and knowledge, patient preferences, patient motivation, patient behaviour | **health and wellbeing** | | | | part of healthcare system. Physiological approach. Pregnancy and birth perceived as life course | complex) groups (teenagers, women with reduced emotional wellbeing, language barrier, caesarean section, infants on NICU, breastfeeding, LGBTQ+, partners, obesity, opiate misuse, pregnancy/neonatal loss. Parenting/ transition to parenthood. Include QaL in quantitative CMC research |
| Capacity for organisational change | Priority of necessary change, monitoring and feedback | | | | | | |
| Knowledge and skills | Domain knowledge, knowledge own practice, skills needed to adhere, | **To emphasise the impact of the midwife's role in society** | 4 | 1 | 2 | *Facilitator* Midwifery perceived as integral part of healthcare system | Research exploring if CMC mitigates social trauma and disadvantage |
| Capacity for organisational change | Mandate, authority, accountability, capable leadership, monitoring and feedback | | | | | | |
| Social, political and legal factors | Economic constraints healthcare budget, contracts, policies, influential people | | | | | | |
| Factors from the CMC evidence | Strength CMC, compatibility, effort, trialability, observability (recommended behaviour) | **To improve the wellbeing of women, families and society** | 3 | 2 | 1 | *Facilitator* Valuing women and midwives, birth, motherhood/ parenthood (society, politics) | Research fundamental knowledge values. Ethical research. Research about (emotional) wellbeing. Include |
| Incentives and resources | (Non)financial (dis)incentives, information system, quality assurance | | | | | | |

| | | | | | | | |
|---|---|---|---|---|---|---|---|
| | and patient safety system, continuing education system | | | | | | QaL in quantitative research |
| Capacity for organisational change | Capable leadership, policies, priority of necessary change, monitoring and feedback | | | | | | |
| Individual health professional factors | Domain knowledge, awareness and familiarity with CMC recommendations, knowledge own practice, skills needed to adhere (knowledge and skills), expected outcome, emotions | **To advocate for continued CMC successes*** | 5 | 2 | 3 | *Barriers* Some health boards have withdrawn from providing CMC. Feeling alone as CMC midwife and/or researcher. CMC not standard model of care *Facilitators* Meaningful conversations with colleagues across Scotland. Connecting with colleagues. Seeing progress of others. Enthusiasm for CMC of others. Discussion with colleagues from other health boards. Collaborative recognition of what is needed. Meeting like-minded people. Share enjoyment. Respect and accept differentiation among midwives (some | Show the positive impact on clinical outcomes and women's experiences. Disseminate evidence of successes/ best practices (CMC newsletter). Continuing dissemination. evidence. Personal stories. Woman/midwife dyad research. Report what works for whom |
| Capacity for organisational change | Capable leadership, relative strength supporters and opponents, priority of necessary change, monitoring and feedback | | | | | | |

| | | | | | | | |
|---|---|---|---|---|---|---|---|
| | | | | | | things work better for some then for others) | |
| Patient factors | Patient beliefs and knowledge, patient preferences, patient motivation | **To safeguard the future of midwives and midwifery** | 4 | 2 | 2 | *Barriers* Skills deficit. *Facilitators* CMC placements. Home birth experience. Continuity mentor-student relationship. Willingness midwives. Government support. | Debate current landscape of maternity services in research. Economic research CMC |
| Professional interactions | communication and influence, team processes, | | | | | | |
| Availability resources | availability resources, incentives, information system, quality assurance and patient safety system, continuing education system | | | | | | |
| Capacity for organisational change | Mandate, authority, accountability, capable leadership, relative strength supporters and opponents, regulation, rules, policies, priority of necessary change, assistance organisational changes | | | | | | |
| Social, political and legal factors | Economic constraints health care budget, contracts, funder policies, | | | | | | |

| | | | | | | | |
|---|---|---|---|---|---|---|---|
| | malpractice liability, influential people | | | | | | |
| Factors from the CMC evidence | Quality of CMC evidence, strength CMC evidence, effort | **To act with integrity** | 3 | 1 | 2 | *Facilitators* Criticize/ challenge patriarchal and technocratic ideologies in research. Debate oppression and subjugation of childbearing women and midwives. Feminism | Position papers. Discussion papers. |
| Professional interactions | Communication and influence | | | | | | |
| Capacity for organisational change | Capable leadership, relative strength of supporters and opponents, monitoring and feedback | | | | | | |
| Factors from the CMC evidence | Quality CMC evidence, strength CMC evidence, clarity, cultural appropriateness, source CMC evidence/ recommendation, consistency with other CMC evidence, feasibility CMC recommendation, compatibility, effort, trialability, observability | **To ensure and sustain the CMC model*** | 5 | 3 | 3 | *Barriers* Reservation about practical reality. Lack of (governmental/ health board) support in model utilisation. CMC not standard model of care *Facilitator* Government support | Evaluate what has/has not worked over past decade and understand current research gaps (health boards). National surveys. Target individual health boards. PROMs and PREMs. Continuing research/ continuing providing evidence. Economic research CMC. Collect evidence from non-early adopters (midwives NHS boards), what is needed to move forward. Disseminate evidence of progress. Disseminate new |
| Individual health professional factors | Domain knowledge, awareness and familiarity with CMC recommendations, knowledge own practice, skills needed to adhere | | | | | | |
| Incentives and resources | (Non)financial (dis)incentives, information system, quality assurance and patient safety system, continuing education system | | | | | | |

| | | | | | | | |
|---|---|---|---|---|---|---|---|
| Capacity for organisational change | Mandate, authority, accountability, capable leadership, relative strength supporters and opponents, regulation, rules, policies, priority of necessary change, assistance organisational changes | | | | | | dissemination plan based on newest Evidence. CMC newsletter to disseminate evidence |
| Social, political and legal factors | Economic constraints health care budget, contracts, funder policies, malpractice liability, influential people | | | | | | |
| Factors from the CMC evidence | Quality CMC evidence, strength CMC evidence, clarity, cultural appropriateness, source CMC evidence/ recommendation, consistency with other CMC evidence, feasibility CMC recommendation, compatibility, effort, trialability, observability, | **To foster a dynamic CMC research culture*** | 5 | 2 | 3 | *Facilitators* Empowerment of women. Feminism | |
| Individual health professional factors | Domain knowledge, awareness and familiarity with CMC recommendations, knowledge own practice, agreement with CMC evidence, attitude towards CMC evidence/ recommendation, intention and motivation, self-efficacy, learning style, | | | | | | |

| | |
|---|---|
| | emotions, capacity to plan change, self-monitoring or feedback |
| Incentives and resources | (Non)financial (dis)incentives, information system, quality assurance and patient safety system, continuing education system |
| Capacity for organisational change | Mandate, authority, accountability, capable leadership, relative strength supporters and opponents, regulation, rules, policies, priority of necessary change, assistance organisational changes, |
| Social, political and legal factors | Economic constraints health care budget, contracts, funder policies, malpractice liability, influential people |

[1] Impact: The likely impact of the unit of significance on the CMC research strategy (the impact if the unit of significance would be part of the mission and/or vision and thus research strategy)

1 = No
2 = Probably not
3 = Uncertain
4 = Probably
5 = Yes

[2] Adherence: The likely impact of adherence to the unit of significance on the CMC research strategy (the level of seriousness of not including the unit of significance in the mission and/or vision/ non-adherence to the unit of significance)

1 = minor impact on adherence
2 = moderate impact on adherence
3 = major impact on adherence

[3] Feasibility: The feasibility of the unit of significance in the targeted settings (how likely will research related to the unit of significance contribute to real-world CMC)
1 = low
2 = moderate
3 = high

**Overview/ Summary barriers and facilitators CMC research mission per TDF domain $Q_3$ Units of Significance**

The barriers and facilitators affecting the implementation of future CMC research align with the research mission using the Theoretical Domains Framework (TDF) to categorise. The TDF framework consists of 12 domains: knowledge, skills, professional role and identity, beliefs about capabilities, optimism, beliefs about consequences, memory, attention and decision processes, environmental context and resources, social influences, emotion, behavioural regulation and goals (Cane et al., 2012).

| Domain | Facilitators | Barriers |
|---|---|---|
| Knowledge | - | Theory-practice gap CMC |
| Skills | Reflective practice | - |
| Professional role and identity | Placing value on physiology on value-based care | Moving away from the true identity of midwifery and midwives |
| | Having a woman-centred focus, | The dissolving midwifery role |
| | Emphasising and acknowledging the specialised identity of midwives | Prioritisation of the institution/ institutional philosophy |
| Beliefs about capabilities | - | Reservation about practical reality of CMC |
| Optimism | Seeing the progress of others | - |
| Beliefs about consequences | Working within professional boundaries/ true role) | Influx in women wanting to birth outside guidelines and free birth |
| Memory, attention and decision processes | - | Some health boards withdrawing from providing CMC |
| Environmental context and resources | Government support | CMC not being a standard model of care or practice, , midwifery is in crisis, the patriarchal and technocratic ideologies in maternity services being task, money, production, technology, politics orientated |
| | Motivation, willingness, and commitment of all stakeholders | Lacking support and commitment on governmental and health board level |
| | System-wide support of CMC (government, NHS management, midwives) | Remuneration of midwives |
| | CMC being a WHO recommendation | Midwifery is in crisis |

| | | |
|---|---|---|
| | Prioritising women and midwives on the national healthcare agenda and in midwifery services | The patriarchal and technocratic ideologies in maternity services (task, money, production, technology, politics orientated) |
| | NHS management respecting midwives | |
| | Buy-in from medical staff | |
| Social influences | Meaningful conversations with colleagues across Scotland (from other health boards), connecting with colleagues and observing enthusiasm for CMC of others | CMC is not a public health priority |
| | A collaborative recognition of what is needed | Silence/ stigma related to pregnancy, childbirth and women's health |
| | Meeting like-minded people and sharing CMC enjoyment and CMC vision | |
| | Acknowledging the differentiation among midwives (some things work better for some than for others) | |
| Emotion | Believing in CMC | Oppression and subjugation related to CMC |
| | | Loneliness of as CMC midwives/ researchers |
| Behavioural regulation | Feminism, empowering childbearing women | - |
| Goals | - | - |
| Barriers: 19 for 9/13 research mission units of significance<br>Facilitators: 27 for 12/13 research mission units of significance | | |

**Overview/Summary research recommendations related to $Q_3$ Units of Significance**

| | |
|---|---|
| Study methodologies | Impact studies, nationwide surveys, economic research, qualitative research, position and discussion papers |
| Theoretical and philosophical research approaches | Epistemology including ideation and ideologies, concept of woman-centred care, the social positioning theory and professional socialisation to capture the role and identity of the CMC midwife |
| Data collection strategies and measures | Women's personal stories/experiences, woman-midwife dyad stories, Patient Reported Outcome Measures (PROMs) and Patient Reported Experiences Measures, clinical outcomes |
| Study population | Childbearing women (public engagement), (student)midwives, health boards, non-early adopters/ non-early adopter sites) |
| Dissemination and communication | CMC progress success and best practices and implementation plan based on newest evidence, CMC newsletter |
| Research recommendations: 7/13 research mission units of significance | |

REFERENCES


Aakhus, E., Oxman, A.D., Flottorp, S.A., 2014. Determinants of adherence to recommendations for depressed elderly patients in primary care: A multi-methods study. Scandinavian Journal of Primary Health Care 32, 170–179. DOI: 10.3109/02813432.2014.984961

Cane, J., O'Connor, D., Michie, S. (2012) Validation of the theoretical domains framework for use in behaviour change and implementation research. *Implementation Science,* 7, 37. doi: 10.1186/1748-5908-7-37

Dunlop, K.L.A., Keogh, L.A., Smith, A.L., Aranda, S., Aitken, J., Watts, C.G, et al., 2023. Acceptability and appropriateness of a risk-tailored organized melanoma screening program: Qualitative interviews with key informants. PLoS ONE 18 (12), e0287591. https://doi.org/10.1371/journal.pone.0287591

Flottorp, S.A., Oxman, A.D., Krause, J., Musila, N.R., Wensing, M., et al, 2013. A checklist for identifying determinants of practice: A systematic review and synthesis of frameworks and taxonomies of factors that prevent or enable improvements in healthcare professional practice. Implementation Sci 8 (35).
