# Supplementary material for: A continuity of midwifery care research mission, vision and agenda
Source: BMC Health Serv Res. 2026 Jun 9;26:1097. doi: 10.1186/s12913-026-14914-3 (PMC13465298; doi:10.1186/s12913-026-14914-3)
Supplement: Supplementary file 2 [file 12913_2026_14914_MOESM2_ESM.pdf]

Additional file 2. Vision: Overview units of significance, TICD domains & determinants, impact & feasibility scores, barriers & facilitators, and recommendations research strategies

TICD domain	TICD determinant	Unit of significance to which the determinant applies	Likely impact of the unit of significance[1]	Likely impact on adherence to the unit of significance[2]	Feasibility unit of significance[3]	Barriers and facilitators	Recommendation research strategies (e.g. specific clinicians, practices, populations, researchers, methodologies, funds)
Individual health professional factors	Domain knowledge, awareness and familiarity CMC recommendations, knowledge own practice, agreement with CMC recommendation, attitude towards CMC evidence/recommendation, expected outcome, intention and motivation, self-efficacy, nature of behaviour	**Sustainable CMC as best practice and standard model of care***	5	3	3	*Facilitator* Government policy prioritising relational care over intervention. Woman-centred paradigm in maternity services. Prioritising women and midwives. Motivation, willingness, commitment all stakeholders and a system wide support of CMC (government, NHS management, midwives)	Research on macro-level, including government, NMC, RCM, NHS management. Document review
Capacity for organisational change	Mandate, authority, accountability, capable leadership, priority of necessary change						
Social, political and legal factors	Economic constraints healthcare budget, contracts, legislation, influential people						
Factors from the CMC evidence	Strength CMC evidence	**Positive (public) opinion CMC***	5	2	2	*Facilitator* Government policy prioritising relational care over	Wide public dissemination. Use social media, influencers, CMC
Patient factors	Patient needs, patient beliefs and knowledge,						

	patient preferences, patient motivation					interventions. Woman-centred paradigm. Prioritising women and midwives. Midwifery perceived as integral part of healthcare system. Valuing women and midwives, birth, motherhood/ parenthood (society, politics)	newsletter to disseminate findings
Incentives and resources	Information system						
Capacity for organisational change	Priority of necessary change						
Social, political and legal factors	Influential people, political stability						
Individual health professional factors	Domain knowledge, skills needed to adhere, attitude towards CMC evidence						
Factors from the CMC evidence	Quality of CMC evidence, strength CMC evidence, clarity, cultural appropriateness, source CMC evidence/ recommendation, consistency with other CMC evidence, feasibility CMC recommendation, accessibility CMC, effort	**Evidence CMC appropriateness, meaningfulness and effectiveness**	4	2	2		Wide public dissemination
Individual health professional factors	Domain knowledge, skills needed to adhere, attitude towards CMC evidence						
Patient factors	Patient needs, patient beliefs and knowledge, patient preferences, patient motivation						
Factors from the CMC evidence	Quality of CMC evidence, strength CMC evidence, clarity, cultural appropriateness	**Open and positive CMC communication/ constructive dialogues (understanding,**	4	2	3		Wide public dissemination
Incentives and resources	information system, quality assurance and patient						

	safety systems, continuing education system	**adoption, refinement)***					
Capacity for organisational change	Capable leadership, relative strength supporters and opponents, priority of necessary change						
Factors from the CMC evidence	Quality of CMC evidence, strength CMC evidence, consistency with other CMC evidence	**Expand body of evidence***	4	3	2	*Barrier* Lack of research funding *Facilitator* Communication and dissemination CMC evidence	Identify research gaps. Needs assessment midwives/ midwifery workforce: workload, emotional load, FTE, support mechanisms, regional differences, team differences (e.g. surveys). Economic evaluation, cost benefit analysis, including QUALYs. Concept analysis. Benchmarking. Comparative study designs: Intervention studies with CoC as the intervention Intervention studies with small team size as the intervention. Theory/ concept development
Factors from the CMC evidence	Quality of CMC evidence, strength CMC evidence, clarity, cultural appropriateness, source CMC evidence/ recommendation,	**Evidence model utility**	4	2	2	*Barrier* Fear and anxiety midwives, obstetricians, management	Needs assessment midwives/ midwifery workforce: workload, emotional load, FTE, support mechanisms, regional differences,

	consistency with other CMC evidence, feasibility CMC recommendation, accessibility CMC, effort						team differences (e.g. surveys). Participatory research/ Include stakeholders, midwives, students
Individual health professional factors	Domain knowledge, skills needed to adhere, attitude towards CMC evidence/recommendation (cognition)						
Capacity for organisational change	Mandate, authority, accountability, capable leadership, relative strength of supporters and opponents, regulations, rule, policies, priority of necessary change, monitoring and feedback						
Social, political and legal factors	Economic constraints on the healthcare budget, influential people						
Individual health professional factors	Domain knowledge, awareness and familiarity with CMC, knowledge own practice, skills need to adhere, intention and motivation, self-efficacy, expected outcome, self-monitoring or feedback, nature behaviour	**Evaluation of the CMC model***	4	3	3	*Facilitator* Institutional/ NHS health board readiness	Needs assessment midwives/ midwifery workforce: workload, emotional load, FTE, support mechanisms, regional differences, team differences (e.g. surveys). Economic evaluation, cost benefit analysis, including QUALYs
Patient factors	Patient needs						
Capacity for organisational change	Mandate, authority, accountability, capable leadership, relative strength of supporters and opponents, regulations, rules, policies, priority of						

	necessary change, monitoring and feedback						
Individual health professional factors	Capacity to plan change (professional behaviour)	**Evidence model efficiency***	5	2	2	*Facilitator*<br>Governmental funding for CMC implementation/ support<br>*Barrier*<br>Strong focus on RCTs	Economic evaluation, cost benefit analysis, including QUALYs. Needs assessment midwives/ midwifery workforce: workload, emotional load, FTE, support mechanisms. Concept analysis. Benchmarking. Comparative study designs: Intervention studies with CoC as the intervention Intervention studies with small team size as the intervention. Is there an argument for looking at how the two models should/could work alongside each other – traditional vs continuity model
Incentives and resources	Availability necessary resources, information system						
Capacity for organisational change	Quality assurance and patient safety systems, capable leadership, relative strength supporters and opponents, priority of necessary change, monitoring and feedback						
Social, political and legal factors	Economic constraints healthcare budget, influential people						
Factors from the CMC evidence	Quality CMC evidence, strength CMC evidence, clarity, cultural appropriateness, source recommendation, consistency with other CMC evidence, feasibility CMC recommendation, accessibility CMC, effort	**Evidence model feasibility***	5	3	3		Asking midwives for their views and opinions. Needs assessment midwives/ midwifery workforce: workload, emotional load, FTE, support mechanisms

Individual health professional factors	Domain knowledge, skills needed to adhere, attitude towards CMC evidence/ recommendation,						
Capacity for organisational change	Mandate, authority, accountability, capable leadership, relative strength of supporters and opponents, regulations, rule, policies, priority of necessary change, monitoring and feedback						
Social, political and legal factors	Economic constraints on the healthcare budget, influential people						
Professional interactions	Communication and influence, team processes						
Incentives and resources	(Non)financial (dis)incentives						
Individual health care practitioner	Emotions, nature of behaviour, self-monitoring or feedback	**Solidarity among midwives**	4	2	2	*Facilitator* Supervision, pastoral support, coaching	Needs assessment midwives/ midwifery workforce: workload, emotional load, FTE, support mechanisms. Participatory research/ Include stakeholders, midwives, students. Feministic research design, give midwives a voice
Professional interactions	Communication and influence, team processes						
Incentives and resources	(Non)financial (dis)incentives, capable leadership, relative strength of supporters and opponents, assistance for organisational changes						
Factors from the CMC evidence	Strength CMC evidence, source CMC recommendation	**Effective mid-level leadership***	5	2	3	*Facilitator* Listening to midwives and women, bottom-up approach within NHS, institutional	Needs assessment midwives/ midwifery workforce: workload, emotional load, FTE,
Professional interactions	Communication and influence, team processes						

Incentives and resources	(Non)financial (dis)incentives, information system, assistance for clinicians					readiness. Managers showing an interest in CMC midwives	support mechanisms. Give midwives a voice
Capacity for organisational change	Mandate, authority, accountability, capable leadership, relative strength of supporters and opponents, regulations, rules, policies, priority of necessary change, assistance for organisational changes						
Individual health professional	Domain knowledge, awareness and familiarity with CMC evidence/recommendation, knowledge own practice, skills needed to adhere, agreement with CMC recommendation, expected outcome, intention and motivation, self-efficacy	**CMC motivation among midwives***	5	3	3	*Barrier* Offering CMC evidence to a profession being in constant turmoil and instability. *Facilitator* Being listened to by mid-level management and non-CMC colleagues.	Study populations of student midwives, newly qualified midwives, midwives with caring responsibilities
Incentives and resources	(Non)financial (dis)incentives, information system, continuing education system, assistance for clinicians						
Capacity for organisational change	Capable leadership, relative strengths supporters and opponents, priority necessary change, monitoring and feedback, assistance for organisational change						

Professional interactions	Communication and influence, team processes	**Midwifery collegiality among CMC and non-CMC midwives**	4	2	3		Participatory research/ Include stakeholders, midwives, students
Incentives and resources	availability necessary resources, incentives, assistance for clinicians						
Capacity for organisational change	capable leadership, relative strength of supporters and opponents, assistance for organisational changes						
Individual health professional factors	Domain knowledge, awareness and familiarity CMC, knowledge own practice, expected outcomes, emotions	**Identity statement CMC midwifery/ midwives***	5	3	3	*Barrier* The midwifery profession being in constant turmoil and instability. Prioritising the institution/ institutional philosophy. Patriarchal and technocratic ideologies. Moving away from the true identity of midwifery/midwives. NMC Standards. Lack of shared vision. Unsupportive CMC workplace culture *Facilitator* Working within professional boundaries/ true role. CMC education. Focus on CMC values. Emphasize and acknowledge the unique identity of CMC midwives.	Research focusing on belief systems. Participatory research/ Include stakeholders, midwives, students. Position statement; use of professional identity theory and theory of (role) transformation. Theory/ concept development
Professional interactions	Communication and influence, team processes						
Capacity for organisational change	Mandate, authority, accountability, relative strength supporters and opponents, regulation, rules, policies, priority of necessary change						

						Placing value on physiology	
Professional interactions	Communication and influence, team processes, resources	**Reflective practice**	4	2	2	*Barrier* Midwifery in crisis. Fear of disciplinary processes (midwives) when preferring CMC or voicing positive opinion. *Facilitator* Willingness CMC	Needs assessment midwives/ midwifery workforce: workload, emotional load, FTE, support mechanisms; Document/historical review
Incentives and resources	Availability necessary resources, information system						
Capacity for organisational change	Priority of necessary change						
Factors from the CMC evidence	Strength CMC evidence	**Research autonomy of midwives/ midwifery CMC domain***	5	3	2	*Barrier* Patriarchy and hierarchy in research domains (medical vs midwifery). *Facilitator* Research skills and education. Research funding. Feminism. Being up to date with political agenda. CMC prioritised on political agenda	
Incentives and resources	Availability of necessary resources, (non)financial (dis)incentives, information system, continuing education system						
Capacity for organisational change	Priority of necessary change, monitoring and feedback						
Incentives and resources	Availability of necessary resources, financial incentives	**Renumeration midwives**	3	3	3	*Facilitator* Feminism	
Capacity for organisational change	Capable leadership, regulation, rules, policies, monitoring and feedback						
Social, political and legal factors	Economic constraints of healthcare budget, contracts, policies, payer, influential people						
Factors from the CMC evidence	Strength CMC recommendation	**(Lifelong) CMC education***	4	2	3	*Facilitator* Educators involved in CMC	

Incentives and resources	Availability necessary resources, (non)financial (dis)incentives, information system, quality assurance and patient safety systems, continuity education system					research.<br>Educators and midwives with PhD (in CMC)	
Capacity for organisational change	Capable leadership, policies, priority of necessary change, monitoring and feedback						
Factors from the CMC evidence	Compatibility, effort, trialability, observability	**Paradigm transformation/ change**	4	2	2	*Barrier* Indoctrination into standard models of care<br>*Facilitator* Shared vision. Believing in CMC	Research focusing on belief systems.<br>Participatory research/ Include stakeholders, midwives, students.<br>Position statement.<br>use of professional identity theory and theory of (role) transformation.<br>Theory/ concept development
Individual health professional factors	Domain knowledge, awareness and familiarity with CMC, knowledge own practice, skills needed to adhere (knowledge and skills)						
Capacity for organisational change	Capable leadership, relative strength supporters and opponents, priority of necessary change, monitoring and feedback						
Factors from the CMC evidence	Quality of evidence supporting the CMC recommendation, strength CMC evidence, clarity, cultural appropriateness, source CMC recommendation, consistency with other CMC evidence, feasibility CMC recommendation	**Evidence long term outcomes maternal health**	4	3	2		Patient reported experiences<br>Other methods than RCT to establish effects on outcomes<br>Include long-term outcomes<br>Include QUALYs<br>Develop tool/ validate tools to effectively measure CoC; Move
Individual health professional factors	Domain knowledge, awareness and familiarity						

	with CMC, knowledge own practice, expected outcomes, emotions						away from single clinical outcomes
Patient factors	Patient beliefs and knowledge, patient preferences, patient motivation						
Incentives and resources	Quality assurance and patient safety systems, continuing education system						
Social, political and legal factors	Economic constraints health care budget, legislation, funder policies						
Factors from the CMC evidence	Quality of CMC evidence, strength CMC evidence, effort,	**Evidence model meaningfulness***	5	3	2	*Facilitator* Feminism	Listen to women's experiences
Patient factors	Patient needs, patient beliefs and knowledge, patient preferences, patient motivation, patient behaviour,						
Capacity for organisational change	Monitoring and feedback						

[1] Impact: The likely impact of the unit of significance on the CMC research strategy (the impact if the unit of significance would be part of the mission and/or vision and thus research strategy)

1 = No
2 = Probably not
3 = Uncertain
4 = Probably
5 = Yes

[2] Adherence: The likely impact of adherence to the unit of significance on the CMC research strategy (the level of seriousness of not including the unit of significance in the mission and/or vision/ non-adherence to the unit of significance)

1 = minor impact on adherence
2 = moderate impact on adherence

3 = major impact on adherence

[3] Feasibility: The feasibility of the unit of significance in the targeted settings (how likely will research related to the unit of significance contribute to real-world CMC)
1 = low
2 = moderate
3 = high

Overview/ Summary barriers and facilitators CMC research mission per TDF domain Q_3 Units of Significance

The barriers and facilitators affecting the implementation of future CMC research align with the research mission using the Theoretical Domains Framework (TDF) to categorise. The TDF framework consists of 12 domains: knowledge, skills, professional role and identity, beliefs about capabilities, optimism, beliefs about consequences, memory, attention and decision processes, environmental context and resources, social influences, emotion, behavioural regulation and goals (Cane et al., 2012).

Domain	Facilitators	Barriers
Knowledge	CMC education, research education, researchers being up to date with the political agenda	-
Skills	Research skills, educators involved in CMC research	-
Professional role and identity	Emphasis and acknowledgement of the unique identity of CMC midwives	-
	Placing value on physiology, focus on CMC values	-
Beliefs about capabilities	Educators and midwives with a (CMC topic-related) PhD	-
Optimism	bottom-up approach in NHS	-
Beliefs about consequences	Working within professional boundaries/ true role	-
Memory, attention and decision processes	-	-
Environmental context and resources	Research funding	Offering CMC evidence to a profession being in constant turmoil and instability, patriarchy and hierarchy in research domains (medical *vs* midwifery)
	CMC prioritised on political agenda,	Lack of research funding
	Motivation, willingness, commitment all stakeholders, system-wide support of CMC (government, NHS management, midwives)	NMC Standards, unsupportive CMC workplace culture

	Listening to women, prioritising women and midwives	Prioritising the institution/ institutional philosophy, patriarchal and technocratic ideologies, a lack of shared CMC vision
	Government policy prioritising relational care over interventions	
	Midwifery being perceived as an integral part of the healthcare system	
	Institutional/ NHS health board CMC readiness	
	Governmental funding for CMC implementation and support	
Social influences	Meaningful conversations with colleagues across managers showing an interest in CMC midwives, midwives being listened to by mid-level management and non-CMC practising colleagues	-
Emotion	-	-
Behavioural regulation	feminism, valuing women and midwives, birth, motherhood/ parenthood (society, politics), a woman-centred paradigm in maternity services	Lack of a woman-centred approach and a lack of relationships with women
Goals	communication and dissemination of CMC evidence	Focus on randomised control trials
Barriers: 11 for 4/13 research vision units of significance<br>Facilitators: 26 for 11/13 research vision units of significance		

Overview/Summary research recommendations related to Q_3 Units of Significance

Study methodologies	Surveys, economic evaluation, cost benefit analysis, including QUALYs, concept analysis, comparative study designs: intervention studies with CMC as the intervention, intervention studies with small team size as the intervention, participatory research, document review, benchmarking
Research approaches	Theory/ concept development, use of professional identity theory and theory of (role) transformation
Research focus	Midwives' belief systems and values, institutional readiness, argument for CMC and non-CMC consisting alongside, research gaps
Data collection strategies and measures	Discourses of women, needs assessment midwives/ midwifery workforce e.g. workload, emotional load, FTE, support mechanisms, regional differences, team differences
Study population	Macro-level, including government, NMC, RCM, NHS management, student midwives, (newly) qualified midwives, midwives with caring responsibilities
Confounders	(Student)midwives' wellbeing, family life and presence/lack of support mechanisms

Dissemination and communication	Position statement, wide public dissemination using social media, influencers, CMC newsletter to disseminate findings
Research recommendations: 11/13 research vision units of significance.	
